# Pituitary suppression with a GnRHa short protocol in an alternate day schedule associated with rhCG microdoses

**DOI:** 10.5935/1518-0557.20140011

**Published:** 2014

**Authors:** Rosane S. Rodrigues, Amanda S. Setti, Daniela P.A.F. Braga, Fernanda M. Valente, Assumpto Iaconelli Jr., Edson Borges Jr

**Affiliations:** 1 Fertility - Centro de Fertilização Assistida, São Paulo, SP, Brazil; 2 Instituto Sapientiae - Centro de Estudos e Pesquisa em Reprodução Assistida, São Paulo, SP, Brazil

**Keywords:** Conservative management, ovarian torsion, uneventful pregnancy

## Abstract

**Objective:**

To evaluate a comfortable short protocol with GnRH agonist (GnRHa) in alternate days, with a step down method of gonadotropins administration associated with hCG microdose for young patients undergoing intracytoplasmic sperm injection.

**Methods:**

This study evaluated 89 ICSI cycles performed in female patients aged <36 years. Patients were submitted to a short protocol with GnRHa schedule in the study group (n= 25) and to a long pituitary suppression protocol in the control group (n=64).

**Results:**

The total dose of rFSH administered as well as estradiol levels on the day of hCG trigger were significantly lower in the short protocol group. There were no significant differences between the groups regarding the fertilization, high-quality embryos, implantation, pregnancy and miscarriage rates. However, mean ovarian stimulation cost (GnRHa short group: $2,397 ± $870.3 and control group: $3,197 ± $658.9, *P* <0.001) and mean ovarian stimulation cost per pregnancy (GnRHa short group: ($4,993 ± $1,813 and control group: $9,743 ± $2,008, *P* <0.001) were significantly lower in the GnRHa short group as compared to the control group.

**Conclusion:**

In patients with normal ovarian response, pituitary suppression with a GnRHa short protocol in alternate days is less costly, requires lower gonadotropins doses and results in similar implantation and pregnancy rates as compared to a GnRHa long protocol.

## INTRODUCTION

The use of gonadotropin-releasing hormone (GnRH) analogues for pituitary suppression in ovarian stimulation protocols was introduced in 1982 (Fleming *et al.*, 1982) and play an important role in in vitro fertilization (IVF) therapy. At this time, there are several alternatives for achieving pituitary suppression in IVF treatments through the use of GnRH agonists (GnRHa) and antagonists.

The GnRHa acts inducing a complete and reversible pituitary suppression by desensitization of the receptors. Therefore, the secretion of follicle-stimulating hormone (FSH) and luteinizing hormone (LH) is suppressed, preventing the premature LH surge and luteinization of follicles and endometrium (Wildt *et al.*, 1986). Long or short protocols as well as daily or single dose administration may be used.

In the long protocol, GnRHa is started in the luteal phase of the previous cycle and continued until the ovulation trigger. The agonist mechanism results in an initial flare of gonadotropin release before the receptors are downregulated. Stimulation with gonadotropin usually starts about 2 weeks later (Hayden, 2008). The use of GnRH agonists in the long protocol is associated with some disadvantages for the patients, such as a long treatment period, the increased risk of the ovarian hyperstimulation syndrome (OHSS), more recurrent manifestation of side effects during the desensitization period (Orvieto & Patrizio, 2013).

In order to decrease costs and side-effects, and enhance practicality, the objective of this study was to test a comfortable short protocol for young patients with GnRHa in alternate days, with a step down method of gonadotropins administration associated with recombinant human chorionic gonadotropin (rhCG) microdoses.

## MATERIAL AND METHODS

### Experimental design, patients and inclusion criteria

This prospective non-randomized study evaluated 89 ICSI cycles performed in a private assisted fertilization centre, between January and December 2012. The inclusion criteria were: Female age less than 36 years, absence of previous ovarian surgery, normal basal FSH and LH levels, body mass index (BMI) inferior to 29 Kg/m^2^, and absence of III- or IV-stage endometriosis. Patients who had not filled these inclusion criteria were excluded from this study.

Patients were submitted to a short protocol with GnRHa schedule in the study group (GnRHa short group, n= 25) and to a long pituitary suppression protocol in the control group (n=64).

Written informed consent was obtained in which the patients agreed to share the outcomes of their cycles for research purposes. The study was approved by the local Institutional Review Board.

### Controlled ovarian stimulation

In the control group, pituitary suppression was achieved by the administration of leuprolide acetate (Lupron Kit; Abbott SA Societé Française des Laboratoires, Paris, France), initiated in the 22nd day of the previous cycle. Controlled ovarian stimulation (COS) was commenced with a daily dose of 225 IU recombinant FSH (rFSH, Gonal F; Serono, Geneva, Switzerland) for a period of 7 days. On day 8 of ovarian stimulation, follicular development was monitored by transvaginal ultrasound and the rFSH dose was reduced to 150 IU until the day of ovulation trigger.

In the short protocol, triptorelin acetate (Gonapeptyl; Ferring, Sao Paulo, Brazil) was initiated in the 1st day of the menstrual cycle, in alternate days. Ovarian stimulation was commenced with a daily dose (day 1 of ovarian stimulation = S1) of 225 IU rFSH (Gonal F; Serono, Geneva, Switzerland), for 3 days. On S4, the recombinant FSH dose was reduced to 150 IU, until the visualization of at least one follicle ≥ 14 mm, when the rFSH dose was reduced to 75 IU and it was concomitantly administered with the recombinant hCG microdose (7.7 µg, equivalent to 200 IU hCG), which was obtained by the dilution of one ampoule of 250 µg of recombinant hCG (Ovidrel; Serono), SC for 2 days. After that, the recombinant hCG microdose was administered alone until the day of ovulation trigger.

When ≥3 follicles attained a mean diameter of ≥17 mm, 250 µg hCG (Ovidrel^®^; EMD Serono, Inc, Rockland, MA, USA) was administered SC. Oocyte retrieval was performed 35 hours later, through transvaginal ultrasonography. The luteal phase was supplemented with a vaginal administration of 90 mg of progesterone gel (Crinone; Serono).

### ICSI and embryo culture, quality and transfer

ICSI was performed according to Palermo *et al.* (1992). High-quality embryos and blastocysts are defined elsewhere (Braga *et al*., 2012).

Embryo transfer was performed on day 5 using a soft catheter with transabdominal ultrasound guidance. One to three embryos were transferred per patient.

### Clinical Follow-up

A pregnancy test was performed 12 days after embryo transfer; a positive pregnancy test was considered to define a biochemical pregnancy. All women with a positive test had a transvaginal ultrasound scan 2 weeks after the positive test; a clinical pregnancy was diagnosed when the foetal heartbeat was detected. Pregnancy rates were calculated per transfer. Miscarriage was defined as pregnancy loss before 20 weeks.

### Data and statistical analysis

The two groups were compared with regard to: (i) total dose of FSH administered, (ii) oestradiol serum levels on the day of hCG trigger, (iii) number of follicles, (iv) number of retrieved oocytes, (v) number of metaphase two (MII) oocytes, (vi) fertilization rate, (vii) percentage of high quality embryos on the third day of development (D3) and (viii) fifth day of development, (ix) number of transferred embryos, (x) implantation rate, (xi) pregnancy rate, (xii) miscarriage rate, and the costs of (xiii) ovarian stimulation and (xiv) per pregnancy.

Data are expressed as mean ± standard deviation for continuous variables, while percentages were used for categorical variables. Mean values were compared by Student’s t test or Mann-Whitney non-parametric test. Percentages were compared by the chi-squared or Fisher exact test, only when the expected frequency was five or fewer. Results were considered to be significant at the 5% critical level (*P* < 0.05). Data analysis was conducted using MINITAB 16 Software.

## RESULTS

There were no significant differences between the GnRHa short group and the control group regarding female age. The total dose of rFSH administered was significantly lower in the GnRHa short group (1,722 ± 691 IU vs. 2,221 ± 521 IU, *P* <0.001). The estradiol serum levels on the day of hCG trigger was significantly higher in the GnRHa short group (3,473 ± 2,763 pg/ml vs. 2,400 ± 1940 pg/ml, *P*= 0.0414). The GnRHa short and control groups showed similar number of follicles, number of retrieved oocytes, mature oocytes, fertilization rate, and high quality embryos rate on day 3 and day 5 of development (Table 1). In the GnRHa short group, 24 patients were submitted to embryo transfer versus 60 in the control group. Two patients in each group had no embryos available for transfer and 3 patients in the control group (4.7%) had ovarian hyperstimulation syndrome (OHSS). The mean number of transferred embryos was similar between the groups (Table 1). Implantation, pregnancy and miscarriage rates were similar between the groups. Finally, mean ovarian stimulation cost was significantly lower in the study group ($2,397 ± $870.3 vs. $3,197 ± $658.9, *P* <0.001), as well as mean ovarian stimulation cost per pregnancy ($4,993 ± $1,813 vs. $9,743 ± $2,008, P < 0.001) (Table 2).

**Table 1:** Comparison of ovarian response to COS and laboratorial ICSI outcomes between GnRHa short and control groups

Variable	GnRHa short group (n=25)	Control group (n=64)	*P-*value
Female age (y-old)	31.5 ± 2.9	31.6 ± 3.1	0.5962
Total dose of rFSH administered (IU)	1,722 ± 691	2,221 ± 521	**<0.001**
Estradiol serum levels on the day of hCG trigger (pg/ml)	3,473 ± 2,763	2,400 ± 1,940	**0.0414**
Number of follicles	17.1 ± 10.6	21.2 ± 13.4	0.1811
Number of retrieved oocytes	13.7 ± 8.6	14.9 ± 9.9	0.6067
Number of mature oocytes	10.5 ± 7.8	10.5 ± 8.1	0.9927
Fertilization rate (%)	73.1	75.5	0.6077
High quality embryos rate on D3 (%)	73.0	70.9	0.6886
High quality embryos rate on D5 (%)	57.5	60.9	0.5361
Cycles transferred (%)	24/25 (96.0)	60/64 (93.8)	0.8542
Mean number of transferred embryos	2.4 ± 0.7	2.3 ± 0.9	0.5414

Note: COS: controlled ovarian stimulation; GnRHa: GnRH agonist; IU: international unit; D3: day 3 of embryo development; D5: day 5 of embryo development.

**Table 2:** Comparison of clinical ICSI outcomes and costs between GnRHa short and control groups

Variable	GnRHa short group (n=25)	Control group (n=64)	*P-*value
Implantation rate (%)	21.5	19.6	0.5501
Pregnancy rate (%)	12/24 (50.0)	21/60 (35.0)	0.2060
Miscarriage rate (%)	3/12 (25.0)	4/21 (19.0)	0.6856
Mean ovarian stimulation cost ($)	2,397 ± 870.3	3,197 ± 658.9	**<0.001**
Mean ovarian stimulation cost per pregnancy ($)	4,993 ± 1,813	9,743 ± 2,008	**<0.001**

Note: COS: controlled ovarian stimulation; GnRHa: GnRH agonist.

## DISCUSSION

This study demonstrated that the GnRHa short protocol, initiated in the first day of the menstrual cycle, in an alternate day schedule, for young patients, results in similar number of follicles and oocytes, and implantation and pregnancy rates as compared to the control group.

In this study we observed a lower total dose of rFSH administered in the GnRHa short group. This happened not only due to the reduction of rFSH dose but also due to the administration of rhCG microdoses. The use of molecules with LH activity in ovarian stimulation was initially proposed with low daily doses of human menopausal gonadotropin (hMG), followed by other studies showing that low doses of rhCG in the late follicular phase are able to complete follicular maturation, even in the absence of rFSH (Filicori *et al.*, 2002, Filicori *et al.*, 2005, Cavagna *et al.*, 2010). Previous studies have showed that the supplementation or substitution of FSH by hCG in the final stages of COS results in a >20% reduction of FSH consumption, yielding a significant cost reduction, without compromising ICSI outcome as compared to traditional COS regimens (Fabregues *et al.*, 2005, Filicori *et al.*, 2005, Cavagna *et al.*, 2010). Therefore, we aimed at evaluating if the GnRHa short protocol in alternate days associated with rhCG microdoses for ovarian stimulation would result in satisfactory IVF outcomes at a reduced cost.

Variations in GnRHa dose appear to be an alternative to decrease the IVF treatment cost. A reduced dose (Out *et al.*, 2000) and an alternate-day regimen of triptorelin (Karatekeli *et al.*, 2006) had no significant effect on ovarian response and the outcome of ICSI cycles. In addition, the alternate-day schedule is interesting because of the reduced number of injections, resulting in a friendlier protocol (Maldonado *et al.*, 2013).

Our results also demonstrated that the study group showed higher serum estradiol levels. We suppose that this happened due to the less profound pituitary suppression achieved with the short protocol and its resulting flare effect. Higher levels of serum LH during the follicular phase after GnRH reduction have been previously observed (Out *et al*., 2000). Moreover, the administration of low-dose hCG alone resulted in a more estrogenic intrafollicular environment (Fabregues *et al.*, 2005). High LH levels might lead to decreased ICSI outcomes (Homburg *et al*., 1988, Loumaye *et al*., 1989, Tarlatzis *et al.,* 1995), however, despite the observation of an increased estradiol levels in the study group we observed no deleterious effect on clinical outcomes with our protocol.

Our findings regarding similar implantation and pregnancy rates corroborate with previous findings (Frydman *et al*., 1988, Acharya *et al.*, 1992). Nevertheless, a randomized prospective study demonstrated a trend towards better implantation and pregnancy rates in the long GnRHa protocol (Ravhon *et al*., 2000).

Regarding ovarian stimulation costs, in this study we observed that the short protocol was more economical; and the cost per pregnancy was significantly lower with this protocol as compared to the long schedule. Maldonado et al (2013) observed that despite the short COS protocol with GnRHa on alternate days associated with rFSH and rhCG microdose may lower the cost of an individual IVF cycle, it requires more cycles to achieve pregnancy. However, in Maldonado’s study, the GNRHa short protocol was compared with the GnRH antagonist protocol. In addition, both the agonist and the antagonist protocols were associated with the administration of hCG microdoses. In the present study, the GNRHa short protocol was compared with the GnRH long protocol, and hCG microdoses were administrated only in the short protocol.

The development of less costly and practical protocols for COS is of pivotal importance, essentially in developing countries in which assisted fertilisation treatments do not qualify for reimbursements. The use of a GnRHa in alternate days associated with rhCG microdoses could be an alternative. Prospective studies with serum LH and progesterone levels assessment are necessary to elucidate the potential negative influence of the flare up effect and estradiol levels on the endometrium.

## CONCLUSION

Our results showed that, in young patients, pituitary suppression with a GnRHa short protocol in alternate days, combined with ovarian stimulation with rFSH and hCG microdoses, requires lower gonadotropins doses, thus, resulting in a more economical approach, without compromising the pregnancy rates, as compared to a GnRHa long protocol.
